# Out-of-body experiences: interpretations through the eyes of those who live them

**DOI:** 10.3389/fpsyg.2025.1566679

**Published:** 2025-04-10

**Authors:** Jenny Moix, Isabel Nieto, Anna Yue De la Rua

**Affiliations:** Department of Basic, Developmental, and Educational Psychology, Autonomous University of Barcelona, Barcelona, Spain

**Keywords:** out-of-body experiences, expanded consciousness, non-local consciousness, phenomenological analysis, experiential interpretation, qualitative research

## Abstract

**Introduction:**

Out-of-body experiences (OBEs) are primarily characterized by the sensation of the self being located outside one's physical body. The complexity of this phenomenon has led researchers to propose various theories to explain it, including physiological, psychological, and non-local consciousness theories. The objective of this study is to directly explore the interpretations of individuals who have experienced this phenomenon firsthand.

**Method:**

The study employed a qualitative descriptive design with a phenomenological interpretive analysis approach, using in-depth semi-structured interviews. The sample comprised 10 participants without mental disorders or neurological and/or vestibular pathologies. The factors studied were predisposing, precipitating, phenomenological, consequential, and interpretive.

**Results:**

All participants agreed that their experience was not only real but described it as more vivid and authentic than everyday reality. Four participants had no explanation for their experience, while one interpreted it in physiological terms. The remaining five explained their experiences using terms like “other planes or dimensions” and “universal consciousness,” aligning with some authors who use concepts such as “non-local” or “expanded consciousness” to address OBEs.

**Discussion:**

The findings suggest that, given that most participants refer to explanations that go beyond what is commonly understood as consciousness, theories of non-local consciousness could be enriched by incorporating these experiential perspectives.

## 1 Introduction

An out-of-body experience (OBE) is defined as a phenomenon in which the center of consciousness appears to temporarily occupy a position spatially remote from one's body (Irwin, 1985). Humans have reported OBEs since ancient times, with descriptions of this complex phenomenon found in most cultures (Metzinger, 2005). Despite the enigmatic nature of OBEs, their occurrence is notable, with an estimated prevalence of 10% to 20% of the population (Alvarado, 2000).

OBEs manifest in diverse ways, with each individual describing unique sensations and circumstances surrounding the experience. The triggering factors are diverse and, in many cases, completely opposite to one another. These experiences can arise in moments of deep tranquility, such as during meditation or relaxation (Alvarado, 1988, 1997; Blanke, 2004; Blanke et al., 2004; Gow et al., 2004; Sellers, 2017; Twemlow, 1989; Wilde and Murray, 2009; Zingrone et al., 2010), or, conversely, in situations of extreme stress, when the individual is in physical danger or undergoing psychological trauma (Alvarado, 1988, 1997; Bateman et al., 2017; Roisin, 2009).

Once the experience is triggered, the sensation of being outside the body can be accompanied by a wide range of emotions. Some individuals describe an absolute sense of peace (Alvarado, 1988; Aujayeb, 2013; Blanke et al., 2004; Gow et al., 2004; Facco et al., 2019; Sellers, 2017; Tressoldi et al., 2015; Weiler and Acunzo, 2024; Wilde and Murray, 2009), while in other cases, the experience can be marked by fear (Blanke et al., 2004; Gow et al., 2004), especially due to the thought: “*I won't be able to return to my body”* (Sellers, 2017).

One aspect that appears to be common among most individuals who have experienced an OBE is the strong conviction that what they are experiencing is real. They do not perceive it as a dream or a hallucination (Alvarado, 1997; Blanke et al., 2004; Campillo-Ferrer et al., 2024; Gallo et al., 2023; Rabeyron and Caussié, 2016; Sellers, 2017; Thakkar et al., 2011; Weiler and Acunzo, 2024; Wilde and Murray, 2009).

In general, OBEs are brief experiences in which the individual feels they are leaving their body, observing themselves from the ceiling, and then returning. However, in some cases, the experience can be much more complex. Some individuals describe traveling to different locations (Alvarado and Zingrone, 1998; Baud, 2017; Sellers, 2017; Tressoldi et al., 2015; Weiler and Acunzo, 2024; Wilde and Murray, 2009) and even encountering other beings (Sellers, 2017; Twemlow, 1989).

Given the complexity and subjective nature of OBEs, analyzing and explaining them within the scientific paradigm is an extremely complex task. It is not surprising, therefore, that the first publications on this phenomenon appeared in parapsychology journals (Alvarado, 1982; De Foe et al., 2013; Irwin, 2000). Over time, the biological and behavioral sciences have begun to investigate these experiences, giving rise to different approaches: psychological, physiological, and non-local consciousness perspectives.

From a psychological perspective, OBEs have been interpreted as a form of mental dissociation, sometimes linked to strategies for coping with traumatic events (Alvarado, 1997, 2016). In contrast, physiological theories suggest that OBEs are distorted perceptions resulting from alterations in the integration of somatosensory and vestibular information (Blanke, 2004; Blanke and Arzy, 2005; Blanke and Mohr, 2005; Blanke et al., 2002, 2004, 2005; Wu et al., 2023). Beyond these approaches, theories have emerged that consider OBEs within the concept of non-local consciousness, moving away from the idea that consciousness is strictly a product of the brain (Brumblay, 2003; Carruthers, 2018; Pace and Drumm, 1992; Persinger, 2010).

Both psychological and physiological approaches have primarily focused on interpreting OBEs as mere consequences of other processes, often overlooking the perspective of those who experience them. The methodologies used in these studies—mainly experimental, quasi-experimental, and observational (analytical or descriptive)—tend to prioritize external interpretations of the phenomenon from researchers' perspectives while neglecting the subjective interpretations of individuals who undergo these experiences.

Following Husserl's (1931) phenomenological framework, we argue that these experiences should be described as they present themselves to consciousness, without resorting to theoretical presuppositions or external causal explanations. This approach emphasizes the value of subjective accounts from those who have experienced OBEs. By analyzing their personal interpretations, we can explore whether OBEs hold intrinsic meaning rather than being mere byproducts of other underlying processes.

Addressing this gap is crucial to achieving a deeper understanding of the phenomenon. This is essential for two main reasons: first, to comprehend the nature of human consciousness, and second, to help normalize these experiences for individuals who undergo them.

In contrast to most studies conducted thus far, this research aims to directly explore the interpretations of individuals who have experienced OBEs firsthand. Moreover, unlike previous studies, this work seeks to provide a comprehensive and systematic understanding of the phenomenon by structuring an analysis through previously defined specific factors. Thus, the goal of this study is to examine predisposing, precipitating, phenomenological, consequential, and interpretive factors, which have been conceptualized as follows:

- Predisposing factors: personality characteristics or psychopathologies that facilitate the experience.
- Precipitating factors: internal or external circumstances that cause the experience.
- Phenomenological factors: the nature of the experience (sensations, emotions, and cognitions).
- Consequential factors: emotional or cognitive repercussions of the experience.
- Interpretive factors: explanations about the reason for the experience.

## 2 Method

### 2.1 Subjects

Sampling was not random due to the specific nature of the study population but was intentional. The inclusion criteria included experiencing at least one OBE and the absence of mental disorders, as well as neuronal and/or vestibular pathologies. Additionally, all participants were functionally independent in their daily lives, either working or studying, and demonstrated clear verbal expression and communication skills during the interviews. Cardiac arrests or other circumstances that cause clinical death or bring the person closer to the end of life can lead to OBEs. In these cases, the name used is: “Near Death Experience” (NDE), which is usually analyzed as a different phenomenon due to its own idiosyncrasy (Klemenc-Ketis, 2013; Van Lommel et al., 2001). Therefore, only subjects who have experienced OBEs in non-near-death situations have been included in the present study.

To mitigate possible biases related to the beliefs of the participants, the sample was not recruited through associations or organizations interested in this phenomenon, but through social networks. Sample size was determined by information saturation and depth of understanding achieved. The final sample consisted of 10 participants, aged between 21 and 56 years, of which 6 were men and 4 women.

### 2.2 Design

The study employed a qualitative descriptive design with a phenomenological interpretive analysis approach (Smith et al., 2009), following the standards proposed by O'Brien et al. (2014). In-depth interviews were conducted to explore individuals' experiences of OBEs and the meaning these hold for them.

### 2.3 Instruments

Information was obtained through semi-structured recorded interviews lasting approximately 1 h. The interview was structured in three phases. In the first phase, structured questions were asked to obtain sociodemographic and quantitative data, such as the number of OBEs and age of onset. The second phase was conducted in a completely open manner, allowing participants to describe in detail their most significant OBE. The third phase consisted of collecting information that had not emerged in phase two about the pre-established categories (predisposing, precipitating, phenomenological, consequential, and interpretive factors).

### 2.4 Procedure

Data collection through interviews was conducted in February 2024. The ethics committee of the Autonomous University of Barcelona (CEEAH protocol 6746) approved this study. All participants provided written informed consent in accordance with IRB-approved procedures. All authors of this article were present during the interviews, though only one was responsible for asking the pre-established questions. The presence of the other authors enabled deeper exploration through additional questions. This dynamic facilitated the capture of useful non-verbal information for subsequent data interpretation.

After completing and transcribing the interviews, the information was extracted and organized. For this extraction, sheets were created with two sections: one for sociodemographic and quantitative data, and another for information on pre-established categories (predisposing, precipitating, phenomenological, consequential, and interpretive factors).

The second and third authors completed the sheets individually. Subsequently, the data were triangulated by comparing the information on the sheets. In cases of discrepancies, these two researchers, together with the first author, discussed the differences until reaching a consensus. Finally, the entire research team analyzed and selected quotes that best represented participants' expressions of the factors. These quotes were included in the results (Tables 1A, B).

**Table 1A:** Predisposing, precipitating, phenomenological, consequential and interpretive factors.

**Participant**	**1**	**2**	**3**	**4**	**5**
Gender	Women	Man	Women	Man	Women
Age	25	36	24	29	56
No. of experiences	1	1	Many	1	Many
Starting age	22	33	20	25	Since childhood
Most meaningful experience	“I was having sex with my ex-partner and it was right at the end. It had an orgasm and right after the experience. I think it was an accumulation of the antidepressants and the orgasm.”	“So, I was working, and I picked up the pallet truck as usual. But something was different, because suddenly I felt like something inside me was trying to get out, like something was rising from me and I started to get scared (...) I started to move quickly the pallet truck so as not to leave my body. At that moment I felt like I was stuck to my ego, to matter.”	“There was one experience in which I felt much more distanced from my physical body (...) I felt that my consciousness and my physical body really took more distance and that if I opened my eyes, I would see my body. At that moment I was scared, so I turned back to my physical body.”	“I was in sleeping in my bed and I had a sensation of separating myself from my body and seeing myself from above. (...) it was like going up of the room, seeing myself sleeping, going up again and no longer seeing me and seeing water. (...) It was above the sea.”	“An aunt of mine (...) had a stroke and they took her to Vall Hebron (Catalan Hospital) I tried to visit her, but I didn't get to where she was (referring to the hospital room). Then, I went to my house and when I was sleeping around 4 or 5 am, I woke up startled because I had seen how I was visiting her in the hospital (...) when I got there I only saw a hallway in the outside area. But in my experience, the doctors let me pass, they made me wash my hands (...) And the next day (...) when I visited her, it was identical. I passed by the same place, I mean, everything was identical. It was like” I've already been here, I was doing this “In that moment I even got a little trembling.”
Phenomenological factors	“It was more relaxation rather than fear. I think it was also because of the previous situation and the orgasm, that's why I wasn't afraid at all. It was weird. I was in a pretty calm and strange state of “*wow what's happening*”. “Yes, I was there, but not a 100%, I was physically in a body, but I was seeing everything from above. The sensation of unfolding.” “It was very real.”	“Yes, it scared me (...) it seemed like I was no longer going to exist in the material world. It is a very strange thing”. “I started not feeling my body. I didn't feel anything. Normally, the pallet truck weighs a lot (...) but when I started having the experience, I did not notice the weigh of it, even my body stopped weighing. “I was so amazed that I was scared.” “This is real, and it is really happening.”	“I stopped feeling like I was in my physical body and my spiritual part was separated”. “More than maybe falling like falling off a cliff, feeling dragged down again.” (describes the return to his body). “When I experience it, I experience it as I am now touching this table, that is, as a reality, as something 100%.”	“I didn't have the feeling of fear.” “It was a sensation that I would define as pleasant, to have another if I could.” “Let's not say pleasant as you are saying, but comfortable, calm.” “I woke up with this feeling that you are falling (...) I woke up suddenly (...) sweating like a chicken.” “And did you perceive it real? -Yes Yes”.	“Like I experience them since I was little, for me it's always been a normal thing”.“It is an experience for me, impressive.” “I think that is when we suddenly enter the body, when we go out to the astral (...) and somehow you have the feeling that you are falling.” “For me it is real, that is, it is normal”, “Have you perceived them as if they were a totally vivid reality? Yes”.
Predisposing factors	Antidepressants.	He did not give any explanation.	She practiced meditation looking for the OBE.	He did not give an explanation.	She believes it is inherited from his maternal grandparents.
Precipitating factors	Orgasm.	Creative meditation through symbols (1st session).	Self-induction with sexual energy transmutation meditation that induces her feelings of tranquility.	Supine position in bed. Calm situation.	Meditations, guided visualizations, relaxation focusing on breathing. Supine position. State of tranquility and security.
Consequential factors	She would experience it again. She explained it only to his partner. His view of reality and his fear of death have not changed.	He would not experience it again, because he was afraid. He explained it to few people, but not because he was worried about the opinion of others. His fear to death did not change.	She would repeat it and currently practice it to achieve a higher level of OBE. She has only told his aunt and close friends. Her worldview has not changed. She is also not afraid of death; she perceives it as a transformation.	He didn't feel afraid, he would repeat it again. He told few friends. He doesn't care if they think he's crazy. His worldview and fear of death have not changed.	Feelings of peace, she considers it a privilege. She shared it with others but for some time she stopped because of her parents' opinion. Her worldview has not changed, since she has had it since she was little, and she is not afraid of death.
Interpretive factors	“More than a soul, a conscience. I don't think it's a soul thing, but like a universal consciousness.” “I already interpreted that way before it happened to me.” “I don't think when you die it's all over.”	“We are not just a body.” “I always think that people in the world, each person has their role in life and I had to experience it to know that it existed and that's it.”	“We are not just a physical body, we all have a spiritual part that many people have asleep or that they do not want to know (...) It's in everyone and the more you work on it, the more aware you become, and the more you will be able to work in it.”	“And I could give you a thousand explanations, such as “*I wanted to feel liberated, and I left”* (...) but I don't think I can explain it. I think it's just something that happens.”	“We are all born with the same abilities (...) but then it depends on each one.” “People who can move on other planes, expanding consciousness or whatever.” “For me it's a privilege, because it makes me understand that there is so much that we do not know (...) you can have a different perception than the one you have every day.”

**Table 1B:** Predisposing, precipitating, phenomenological, consequential and interpretive factors.

**Participant**	**6**	**7**	**8**	**9**	**10**
Gender	Man	Man	Man	Man	Women
Age	28	23	21	28	52
No. of experiences	1	2	1	1	Many
Starting age	17	16	15	21	7
Most meaningful experience	“The group meditation was guiding me and during the process I stopped hearing the man and stopped being where I was. (...) I felt that I wasn't there physically, that I was in another place, like a kind of dark hallway.”	“First I felt a little numb, my body felt heavier, obviously it wasn't my physical body. If I remember correctly, I tried to look at the window.” “It was as if the moonlight was blue (...) I saw the tree and everything blue, the floor, the walls, the environment, if it was blue, I don't know how to describe it, but as if the moon directly dyed everything in blue”.	“I was with a friend on a bench on the street (...) and we stayed in silence for a long time and at that moment I kind of closed my eyes (...) then my point of view went like over my neck (...) And there was a moment that I got scared because I said “*Damn, I'm leaving*” and then I came back.” (...) “And how much did you went up? Well, maybe at the height of a second floor of a building.”	“I have only had one experience and it was taking drugs. (...) And when I took it it made me feel dizzy and I started walking and I saw myself walking. (...) I saw my head and my body ahead and I was a little confused. “It disappeared after five minutes, more or less.”	“For me, the most significant was when I decided to go to Scotland.” (...) when you decide to go somewhere (...) something absorbs you in and you are directly in that place (...) I was looking at the landscapes (...) and I arrived at a village, I still remember it super vivid (...) and I also remember that I kept some names (...) because I wanted to know if it really existed” “And once I returned, it is true that I went to check if the town existed. I saw photos of where I had been, the same photos, the same place in the same cabins, everything.”
Phenomenological factors	“Fear as discomfort.” “I was literally afraid to sleep for half a month.” “I felt like all of me was in the hallway. That's why it was the feeling that it was so real, just like I'm sitting in my chicken coop now.”	“It's like the feeling you get when someone is staring at you, that happened to me with the tree.” “I was curious, because it was something new that I had never seen.” “I felt that I could move as I wanted, up, down, left, right, I only felt tiredness, but a feeling of freedom. I could say that I was a little freer.” “It felt quite tangible.”	“I was scared, because I said “*I'm not coming back to my body*” at that moment it was like “*I'm leaving*”, I don't know, it made me feel scared.” “In my memory it does not have the characteristics of a dream (...) it impacted me a lot and I remember it because it has never happened to me again.” “I remember there was wind when I experienced it, because I really like the breeze and I remember feeling it at that moment, but when I left, I didn't feel it anymore.”	“I was freaking out (...) I didn't feel fear, maybe euphoria.” “I felt like I was walking, I felt like I had to walk at that moment, but it wasn't like it was automatic (...) I just don't know how to describe it, it's very strange to describe it.” “And what you saw, did you perceive it as real? -Yes Yes.”	“When you leave the body (...) you automatically enter what I call astral (...) your consciousness expands and you understand much more of what there is.” “The body is an empty container, but your consciousness remains exactly the same, so the emotions are not contained within what the physical body is, the emotions are contained in your consciousness.” “There is a gigantic feeling of peace.” “Have you experienced it real? Yes, it is something that makes you feel that this life is the one that is really transitory, like an experience within what is true reality.”
Predisposing factors	Interest in the topic and looking for experience.	Lucid dreaming led him to the desire to experience OBEs.	He did not give an explanation.	He did not give an explanation.	My grandmother, great-grandmother, great-great-grandmother, from my maternal side were healers, which made me grow up with a more open mind to the unknown and paranormal, “spiritual” to define it in some way.
Precipitating factors	Group meditation to get started on the OBEs. Position: sitting in a chair.	Self-induction with meditation.	State of tranquility and breeze.	Taking ketamine.	Supine position. State of tranquility and relaxation, otherwise the physical body will not let you separate.
Consequential factors	Very afraid and stopped meditating. He would repeat it to check what happened. He explained it to a few people, but not out of fear or shame. It did not change his view toward death or his way of thinking.	He felt upset. He would repeat it, but it would be difficult for him to blank his mind. He explained it to a friend and her partner, in case people don't believe him. It made him think that we do not die, but rather we transform.	He was afraid, he thought he had died. He would repeat it if he could control it, and he doesn't explain it for fear of people's reaction. It didn't change his worldview.	I would repeat it through meditation. He has told friends and doesn't mind telling it. The OBE made him interested in spiritual matters.	Tiredness, energy exhaustion, spending a lot of energy traveling. She talks about it openly. She is resuming the OBEs because her physical body did not allow her to have them. The OBEs have made her to see that in this reality we worry about things that in the astral we would not.
Interpretive factors	“I guess it was because I was used to meditating, or maybe I was more relaxed than normal, and so I went on a little trip.”	“I would say that it is an experience that helps you a lot to concentrate on yourself, to relax (...). I see it more as a connection with oneself and I find it quite interesting and strong that you can do that.” “I think what would best describe it would be conscience.”	“I'm not going to think “*I have powers*” because I don't.” “I can't give you an explanation, like “*my soul has gone*” “At that moment (...) I thought “*I'm leaving the body, I've died*” “That's why I say that maybe I fell asleep or half asleep, I don't know.”	“I believe that in my experience (...) it was more caused by the drug and a matter of perceptions, rather than an astral issue (...). I guess that for people who practice it usually, it's to have a higher level of consciousness, that is how I would define it. In my case taking drugs… well, look, I wouldn't know what to tell you.”	“Astral travel opens your consciousness (...) it teaches you that this is not the only thing, that there are more dimensions, that it is different, that it is much broader, that there is a much greater truth, which is not the one that is here”. “It has to do with areas of the brain that are activated and are related in some way to our consciousness. And that some people, due to certain situations, are more predisposed to activate these areas than others.”

## 3 Results

The results are found in Tables 1A, B. As can be seen, the age of onset of two participants was in childhood, three in adolescence and five in youth. Of the 10 participantss interviewed, seven have experienced OBEs once or twice in their lives while 3 on numerous occasions.

### 3.1 Most meaningful experience

The results indicate that the experiences are very idiosyncratic, although some common elements were identified. In cases 1, 4, and 7, the participants experienced the out-of-body sensation while lying in bed. Participants 1 and 4 saw their bodies from the ceiling, while participant 7 saw theirs from another part of the room. Participant 8 had the experience while sitting on a street bench and viewed their body from the sky, approximately at the height of a second floor. Participant 6 found himself suddenly in a long, dark hallway. Participant 9 saw his body from behind while walking. Participants 5 and 10 reported traveling to very distant places, such as other cities or countries.

### 3.2 Phenomenology of experience

In cases 2, 3, and 6, the participants felt fear. However, the rest of the participants described their experiences with the following words: calm and strangeness (1), pleasant and comfortable (4), impressive (5), curiosity and freedom (7), euphoria (9), and peace (10). In one case (8), despite living the experience fully, fear arose when thinking that he would not be able to return to the body. All participants perceived the experience as real. The words of participant 3 exemplify this: “I experience it as I am now touching this table, that is, as a reality.”

### 3.3 Predisposing factors

Participant 1 stated that at the time she experienced the OBE, she was taking antidepressants that caused dissociative states, which she believed predisposed her to the experience. Participant 2 mentioned that a few hours before feeling the onset of his OBE, he had performed meditative therapy for the first time and attributed the OBE to this, as it had produced a kind of internal change: “a strange sensation as if something had moved in me.”

For participant 3, the predisposition was her own motivation. Her aunt, who experienced constant OBEs, encouraged her to have them as well. Two of the participants (5 and 10) had experienced OBEs since childhood, which made it difficult for them to identify predisposing factors. However, participant 5 suggested there might be some hereditary influence from her maternal grandparents. Similarly, participant 10 spoke of influence from her maternal side: grandmother, great-grandmother, great-great-grandmother, all of whom were healers. This, according to her, led to her growing up in an environment open to the unknown and spiritual. Participant 7 stated that lucid dreaming led him to seek ways to self-induce OBEs. The other interviewees (4, 6, 8 and 9) did not have any explanation as to what could have predisposed them.

### 3.4 Precipitating factors

Three of the participants (3, 6, and 7) induced their OBEs through meditation, while another participant (2) experienced it because of meditation but did not actively seek to provoke the experience; rather, it occurred unexpectedly. For four participants, the OBEs happened in tranquil settings: while lying in bed (4, 5, and 10) and while sitting on a bench at dusk (8). For participant 1, the OBE was triggered by an orgasm. And for participant 9, it resulted from consuming Ketamine.

### 3.5 Consequential factors

The interviewees were questioned regarding three specific aspects of the experience: (1) whether it left them with a positive impression, prompting a desire to repeat it; (2) if it altered their perspective on death or their worldview; and (3) whether they chose to explain it with others. The question about whether they would repeat the experience only made sense for the seven participants who had experienced it once or twice given that the other three experienced it repeatedly. Among these seven, participant 2 said that he would not repeat it out of fear. In fact, when this individual began to sense the departure from his body, he managed to stop it; it could be argued that he never fully underwent the OBE due to fear. The remaining six participants expressed a willingness to repeat the experience, although some with conditions. Participant 8 would do so if he could exert control over it and participant 9 if it was induced through meditation and not ketamine. Participant 6 stated that, despite the fear it provoked, would repeat it out of curiosity. Finally, three interviewees (1, 4, 7) would repeat it without conditions because they lived it as a very pleasant experience.

When questioned about whether their perspective on death or their worldview had changed, only two participants responded affirmatively. Participant 7 conveyed that the experience led him to believe in a notion of transformation rather than cessation, suggesting that we do not perish; rather, we transform. Participant 10, who had experienced numerous OBEs, reflected that traversing “another plane” had granted her insight into the insignificance of many concerns prevalent in our everyday lives. Those who stated that the experience had not changed them (1, 2, 3, 4, 5, 6 and 9) argued that they already had a spiritual vision, and the experience confirmed it. Participant 8, however, articulated that he maintained his disbelief in anything beyond death both before and after the experience because he considered that the experience was not proof of anything spiritual.

Regarding whether the participants had explained their experience, participant 8 had not told it to anyone for fear of the judgment of others. Participants 1, 3 and 7 had only confided in a single individual or their closest circle for similar reasons. Participant 5 revealed that during childhood, she kept it to herself due to concerns about her parents' reaction. Conversely, the remaining participants (2, 4, 6, 9, and 10) shared their experiences without reservation, unconcerned about others' perceptions.

### 3.6 Interpretative factors

None of the participants claimed to possess a definitive explanation for their experiences, except for the individual who attributed it solely to ketamine (9), suggesting a purely physiological origin. Four of them stated that they had no explanation: “I think it's something that just happens” (4); “it was just a little trip” (6); “maybe I fell asleep or was half asleep, I don't know” (8); and “I had to experience it to know that it existed and that's it” (2). The remainder consistently expressed uncertainty, offering simple hypotheses or possibilities. Explanations varied, including notions of contact with a universal consciousness (1), heightened awareness of the spiritual aspect of the self (3 and 7), and moving in other planes and dimensions (5 and 10).

## 4 Discussion

The present study is based on in-depth interviews with individuals who have experienced OBEs. As a qualitative study, it does not allow for generalizable conclusions. Instead, the objective is to understand the experiences and personal explanations of the phenomenon from the participants' perspectives. Unlike most literature that aims for researchers to reach specific conclusions, this study prioritizes the participants' own hypotheses.

The results of this study confirm that OBEs are very idiosyncratic experiences, both in their phenomenology and in the precipitating and consequential factors (Alvarado, 1982, 1988; Aspell and Blanke, 2009).

Regarding the age of onset, it was observed that the first OBE typically occurred during childhood or youth. However, it is difficult to determine whether OBEs also commonly begin in adulthood, as the sample was predominantly composed of individuals under 30 years old (7 out of 10). Among the two participants over 50 years old, both reported having experienced their first OBE during childhood. According to Alvarado (1988), studies that attempt to correlate the occurrence of OBEs with age generally do not find a significant correlation. However, Blackmore (1986), through a questionnaire administered to 97 individuals, observed significant differences in age between those who reported experiencing OBEs and those who did not. Those who had experienced OBEs tended to be younger.

Most participants (7 out of 10) reported experiencing only one or two OBEs throughout their lives, with just three indicating frequent occurrences. This finding aligns with previous literature (Blanke, 2004; Parra, 2008).

### 4.1 Predisposing factors

Throughout the research on this topic, several researchers have postulated various predisposing factors, including: beliefs (Gow et al., 2004; Parra, 2010, 2018); the absorption capacity (Gow et al., 2004; Irwin, 2000; Parra, 2010, 2018); psychopathologies (Gow et al., 2004; Irwin, 2000; Lopez and Elzière, 2018; Mudgal et al., 2021; Murray and Fox, 2005; Parra, 2010; Roisin, 2009; Terhune, 2006) or neurological pathologies (Blanke, 2004; Blanke and Arzy, 2005; Yu et al., 2018). However, unlike these investigations, the present study aimed to explore predisposing factors based on the participants' own intuitions rather than those posited by researchers. Additionally, to understand their opinions on the researchers' hypotheses regarding this point. In this regard, none of the participants indicated that their personality traits might have predisposed them to experience OBEs. Only the motivation to experience the phenomenon was identified as a predisposing factor, as mentioned by two participants. One participant was taking antidepressants at the time of the experience; however, she attributed the OBE not to depression but to the effects of the medication.

Regarding beliefs, certain authors maintain that beliefs of a spiritual nature can facilitate OBEs to a greater extent than those of a scientific nature (Gow et al., 2004; Parra, 2010, 2018). In line with this hypothesis, one of the participants who had experienced OBEs since childhood mentioned growing up in an environment conducive to paranormal and spiritual phenomena, suggesting that this could have predisposed her. In contrast, the rest of the participants did not indicate that their beliefs had any influence.

The data from this study suggest that, for most participants, their beliefs did not influence their first OBE. When asked to explain their experiences, half of them did not provide any spiritual explanation, instead expressing uncertainty or offering physiological explanations. Other studies have also not found a significant relationship between beliefs and OBEs (Aujayeb, 2013; Bova, 2011; Terhune, 2006; Wilde and Murray, 2009).

Although our data suggests that beliefs do not predispose individuals to their first experience, they do appear to influence its repetition. This assertion is based on the observation that in our sample, the three individuals who experienced numerous OBEs believed in realities beyond our own. While we cannot definitively determine the direction of this influence, it is possible that their mindset has contributed to the increased incidence of OBEs, or perhaps experiencing multiple OBEs has reinforced their beliefs.

If we categorize OBEs based on their complexity, we can distinguish between simple and complex experiences. Simple OBEs involve the participant leaving their body but not moving far from it, remaining in a place where they can observe their physical body. In contrast, complex OBEs involve longer journeys and a variety of experiences. In our research, most participants have experienced simple OBEs, and the vast majority have had only one such experience. Conversely, participants who have experienced more complex OBEs have done so on numerous occasions. This leads us to hypothesize that the complexity of OBEs can increase with repeated experiences.

The experience of lucid dreams has also been linked to OBEs (Blackmore, 1986; Raduga et al., 2020). In line with this association, one participant in our sample habitually experienced lucid dreams. Moreover, he stated that it was precisely the experience of lucidity during sleep that led him to seek out OBEs, suggesting that, for him, the two phenomena were somehow related.

### 4.2 Precipitating factors

Before experiencing OBEs, most participants were in calm situations, supporting findings documented in the literature (Blanke, 2004; Blanke et al., 2004; Gow et al., 2004; Sellers, 2017; Wilde and Murray, 2009; Zingrone et al., 2010). However, we cannot conclude that only these types of circumstances act as precipitating factors, as other studies have also observed that stressful situations can trigger them (Aujayeb, 2013; Bateman et al., 2017; Brandt et al., 2009; Roisin, 2009).

Self-induction through various meditative methods has also been observed as a trigger for OBEs in three of the participants, confirming the findings of other studies (Alvarado, 1982; Blanke and Mohr, 2005; Bova, 2011; Sellers, 2017; Smith and Messier, 2014). Additionally, consistent with Wilkins et al. (2011), this research has demonstrated that Ketamine can act as a precipitating factor.

In one of the participants, orgasm was identified as a precipitating factor. However, we have not found information about this in other investigations. We consider that the physiological alteration induced by the orgasm could have led to the experience. This participant mentioned that the feelings of dissociation caused by the antidepressants she was taking could have predisposed her. Therefore, we hypothesize that the medication may have predisposed her, and that the orgasm triggered the experience.

### 4.3 Phenomenological factors

Numerous investigations have highlighted two prevalent sensations in OBEs: the feeling of peace (Aujayeb, 2013; Blanke et al., 2004; Gow et al., 2004; Facco et al., 2019; Sellers, 2018; Tressoldi et al., 2015; Wilde and Murray, 2009) and the sense of reality (Blanke et al., 2004; Rabeyron and Caussié, 2016; Sellers, 2017; Thakkar et al., 2011; Wilde and Murray, 2009). In line with these findings, all the participants in our sample stated that they felt the experience as real, clearly distinguishing it from a hallucination or a dream; and most described it as full of peace.

Certain research has demonstrated that the experiences participants have during OBEs align with reality. For instance, Ring and Cooper (1999) noted that some individuals blind from birth reported visual perceptions during their OBEs, which were later validated as accurate. Parra (2008) suggests that the verifiability of many OBEs is attributed to some form of extrasensory perception of environments that are physically inaccessible. In our sample, two participants affirmed that reality confirmed what they had experienced during their OBEs. Participant 5 described an out-of-body experience where she visited the hospital to see her aunt in the Intensive Care Unit (ICU). The following day, upon visiting the hospital in reality, she was deeply surprised to find that the hallway, the door, and the ICU where her aunt was located were exactly as she had seen during the OBE. Participant 10 reported that during her OBE, she visited a village in Scotland. As she flew in, she observed a bridge and a specific landscape, and upon “landing” in the village, she noticed the village's name. Later, she confirmed on a map that both the river and the village existed. From the subjective perspective of these participants, there seems to be a clear correspondence between their experiences during the OBE and reality. Nevertheless, it is crucial to note that these cases do not allow us to draw objective conclusions in this regard.

The presence of fear has also been observed in some cases, especially related to the feeling of lack of control and concern about being able to return to one's own body. These sensations have been documented in other studies (Sellers, 2017).

### 4.4 Consequential factors

Some studies show that people who experience OBEs decrease their fear of death (Shaw et al., 2023; Irwin, 1988; Osis, 1979). In our sample, only one participant explained that the experience made her feel that death is just a transformation. It was found that people who did not believe in anything else after death still did not believe it after the experience. In fact, Irwin (1985) and Palmer (1979) maintain that OBEs do not typically produce religious conversions among atheists. Conversely, the more spiritual participants maintained that the OBEs only reaffirmed their previous beliefs.

We have noted that people who experience OBEs occasionally do not suffer major changes in their lifestyle or interpretation of life. This finding contrasts with participants from other studies, such as that of Shaw et al. (2023), where beneficial changes have been observed after OBEs. We believe that the impact of the experience may depend on its content and the individual's own characteristics. However, it was observed that the two participants who experienced the phenomenon since their childhood had a perspective on life where the problems were relativized to the maximum, confirming what was found in other studies (Baud, 2017; Bova, 2011; Rabeyron and Caussié, 2016; Wilde and Murray, 2009, 2010) and in which there was no fear of death (Shaw et al., 2023). It seems that the beneficial effects occur mainly in people who have had the experience on numerous occasions. In fact, in the study by Shaw et al. (2023), where profound changes have been observed as a result of OBEs, most participants in the sample had experienced them repeatedly. In these cases, we could say that OBEs can be considered transformative experiences, as defined by many researchers: as phenomena able to engender long-lasting, irreversible, pervasive consequences on individuals' beliefs, perceptions, identity, and values (Chirico et al., 2022).

According to the literature, most people who experience the phenomenon would like to repeat it (Gabbard and Twemlow, 1984; Irwin, 1988; Osis, 1979); we have found the same tendency in the participants of our sample.

In this research, some people opted not to share their OBE with anyone or only confided in those close to them, fearing they would not be understood or labeled as disordered. From this arises the importance of conducting outreach efforts to familiarize the general public with this phenomenon and foster its normalization, as other authors also suggest (Bova, 2011; De Foe, 2012). This participant not only requires dissemination among the general public but is also imperative for inclusion in the training curriculum of health professionals. It is crucial that they receive adequate training to effectively assist individuals who have undergone this phenomenon. The absence of understanding regarding this topic could result in incorrect diagnoses and difficulties in its management by healthcare professionals, as highlighted by De Foe (2012).

### 4.5 Interpretative factors

How do the participants themselves interpret the OBE phenomenon? Some simply confess that they have no explanation. This lack of explanation is also found in the scientific field. Despite countless investigations in this regard, there are no solid conclusions, but rather a multitude of hypotheses from vastly different paradigms.

One participant attempted to explain the phenomenon in physiological terms, as a state resulting from ketamine. Many authors try to explain OBEs in physiological terms. From a biological framework, they are conceived as distorted perceptions resulting from a failure in somatosensory and vestibular integration (Blanke, 2004; Blanke and Arzy, 2005; Blanke and Mohr, 2005; Blanke et al., 2002, 2004, 2005; Bos et al., 2016). The fact that some people with neurological disorders, such as epilepsy or headaches, present OBEs corroborates this view (Blanke, 2004; Blanke and Arzy, 2005; Blanke and Mohr, 2005; Blanke et al., 2002, 2004; De Ridder et al., 2007; Fang et al., 2014).

Dissociation has been proposed as a psychological explanation for this phenomenon, as many individuals appear to undergo the experience to evade a traumatic event (Alvarado, 2016; Bateman et al., 2017; De Ridder et al., 2007; Gow et al., 2004). This dissociation, as pointed out by Parra (2008), does not signify any psychopathology, contrary to historical assumptions (Alvarado, 1992; Facco et al., 2019); rather, it is viewed as a beneficial coping strategy. However, in this study, none of the participants reported their experience as dissociative. Moreover, none of them were in a stressful situation necessitating dissociation to alleviate distress.

In conventional scientific terms, consciousness is a result of evolution, the biological adaptation of the nervous system. Consciousness is viewed as an individual phenomenon within this framework. In contrast to this traditional understanding of consciousness, half of the participants in our study used terms such as “dimensions”, “planes”, “universal consciousness”, among others. Their expressions were aligned with emerging theories on expanded or non-local consciousness. According to these theories, the mind or consciousness is not completely confined to the brain or body but may have properties that transcend space and time (Hameroff and Penrose, 2014; Tononi and Koch, 2015). The concept of nonlocality finds support in quantum physics, which explores how subatomic particles separated by vast distances can be entangled in a manner beyond explanation by conventional physics. Some authors approach OBEs from this novel perspective (Brumblay, 2003; Pace and Drumm, 1992; Persinger, 2010).

### 4.6 Final considerations: relevance, limitations and future research avenues

As highlighted by Shaw et al. (2023), most research in this domain tends to be correlational, seeking connections between personality traits or pathologies and OBEs, or concentrating on individuals with neurological disorders. Researchers typically attempt to elucidate this phenomenon through psychological and/or physiological hypotheses. In contrast, our study did not start with an explanatory hypothesis; rather, it aimed to explore the interpretations of the participants themselves. This approach enabled us to compare the perspectives of researchers observing the phenomenon from an external standpoint with the interpretations of participants experiencing it firsthand.

None of the participants in our sample considered their experience to be a hallucination or a perceptual error, as some theories suggest. Even the participant who experienced OBEs under the effects of ketamine stated that what he experienced was real. Likewise, none of them related the experience to any trait of their personality. When some of the participants discussed the potential involvement of their brains in OBEs, they did not interpret it as a disorder or a failure in information integration, as some physiological theories suggest. Instead, they perceived it as an amplification or augmentation of their perception or experiential abilities.

Qualitative studies should persist in capturing the interpretations of the participants themselves and the phenomenology of their experiences. Nonetheless, such studies have their limitations. One of these is information saturation, used as a parameter to determine sample size. Given the complexity of such phenomena, and the necessity for obtaining highly detailed information, achieving saturation is exceedingly complicated; it would require such a large sample size that conducting in-depth analysis would become difficult.

One strategy to mitigate this limitation is to delimit, to the greatest extent possible, the objective of the study. In our case, we have analyzed multiple factors (predisposing, precipitating, phenomenological, consequential, and interpretive). However, in future qualitative studies, it would be more appropriate to focus on a single factor for deeper analysis. Following the example of Shaw et al. (2023), who focused solely on the transformative aspects of OBEs, could be beneficial.

Another way to address this limitation is to ensure a more homogeneous sample. Specifically, we propose to homogenize the number of previous OBEs. In our study, most participants had experienced the phenomenon only once in their lives, while two individuals had experienced it since childhood. This difference was notable in how they interpreted the experience and its impact on their lives. Therefore, we recommend investigating these two groups separately. In line with this proposal, we find Neppe's (2011) perspective. He suggests that the diversity of theoretical perspectives on OBEs is due to each model likely examining a specific type of OBE. Therefore, he argues that the correct approach would be to describe OBEs from a detailed phenomenological perspective to classify them appropriately. This would allow for a better understanding of these experiences.

The future of research in this field can follow two main paths: applied and basic research. In terms of applied research, we believe the initial goals should focus on two key aspects: normalizing this phenomenon and fostering personal growth and self-awareness in those who experience it.

Normalization is essential, as our study confirms that most individuals hesitate to share their experiences out of fear of being perceived as mentally disturbed. Additionally, our findings support the idea that OBEs can, in some cases, lead to profound personal transformations. Therefore, enhancing and integrating these experiences could also have therapeutic potential.

Moreover, beyond these objectives, there is an opportunity to gain valuable insights from individuals who, through their OBEs, have developed a more relative perspective on life and have overcome the fear of death. These insights could be instrumental in addressing psychological conditions associated with existential distress.

And within basic research, the study of OBEs would allow us to explore the concept of consciousness more broadly. By viewing the OBE phenomenon as something natural and not something strange to be explained, we could draw on the experiences and interpretations of the people who experience it to arrive at unexplored points of view about consciousness. The current scientific paradigm appears too narrow to adequately address the complexity of this phenomenon. Adopting an open-minded approach could aid in integrating various perspectives, from physiological and psychological to non-local consciousness, which need not be mutually exclusive. This could lead to a more comprehensive understanding of consciousness.

## Data Availability

The raw data supporting the conclusions of this article will be made available by the authors, without undue reservation.

## References

[B1] Alvarado C.S. (2016). Out-of-body experiences during physical activity: report of four new cases. J. Soc. Psychical Res. 80, 1–12.

[B2] Alvarado C. S. (1982). ESP during out-of-body experiences: a review of experimental studies. J. Parapsychol. 46, 209–230.

[B3] Alvarado C. S. (1988). Psychological aspects of out-of-body experiences: a review of spontaneous case studies. Puerto Rican J. Psychol. 5, 31–43.

[B4] Alvarado C. S. (1992). The psychological approach to out-of-body experiences: a review of early and modern developments. J. Psychol. 126, 237–250. 10.1080/00223980.1992.10543358

[B5] Alvarado C. S. (1997). Mapping the characteristics of out-of-body experiences. J. Am. Soc. Phys. Res. 91, 27–32.

[B6] Alvarado C. S. (2000). “Out-of-body experiences,” in Varieties of Anomalous Experience: Examining the Scientific Evidence, eds. E. Cardeña, S. J. Lynn, and S. Krippner (Washington, DC: American Psychological Association), 183–218. 10.1037/10371-006

[B7] Alvarado C. S. Zingrone N. L. (1998). A study of the features of out-of-body experiences in relation to Sylvan Muldoon's claims. J. Parapsychol. 62:104.

[B8] Aspell J. E. Blanke O. (2009). “Understanding the out-of-body experience from a neuroscientific perspective,” in Psychological scientific perspective on out-of-body and near-death experiences, ed. C. D. Murray (New York: Nova Science Publishers, Inc), 73–88.

[B9] Aujayeb A. (2013). Out-of-body experience in the Karakorum. Wilderness Environ. Med. 24, 295–297. 10.1016/j.wem.2013.01.012 23541594

[B10] Bateman L. Jones C. Jomeen J. (2017). A narrative synthesis of women's out-of-body experiences during childbirth. J. Midwifery Women's Health 62, 442–451. 10.1111/jmwh.12655 28731565

[B11] Baud S. (2017). Expériences ≪ hors du corps ≫: un voyage ≪ en esprit ≫ à la rencontre d'un autre de soi. Intellectica 67, 347–366. 10.3406/intel.2017.1849

[B12] Blackmore S. J. (1986). Spontaneous and deliberate out-of-body experiences: a questionnaire survey. J. Soc. Psychical Res. 53, 218–224.

[B13] Blanke O. (2004). Out-of-body experiences and their neural basis. Br. Med. J. 329, 1414–1415. 10.1136/bmj.329.7480.1414 15604159 PMC535951

[B14] Blanke O. Arzy S. (2005). The out-of-body experience: disturbed self-processing at the temporo-parietal junction. Neuroscientist: A Rev. J. Bringing Neurobiol. Neurol. Psychiatry 11, 16–24. 10.1177/1073858404270885 15632275

[B15] Blanke O. Landis T. Spinelli L. Seeck M. (2004). Out-of-body experience and autoscopy of neurological origin. Brain 127, 243–258. 10.1093/brain/awh040 14662516

[B16] Blanke O. Mohr C. (2005). Out-of-body experience, heautoscopy, and autoscopic hallucination of neurological origin. Brain Res. Rev. 50, 184–199. 10.1016/j.brainresrev.2005.05.008 16019077

[B17] Blanke O. Mohr C. Michel C. M. Pascual-Leone A. Brugger P. Seeck M. Landis T. Thut G. (2005). Linking out-of-body experience and self processing to mental own-body imagery at the temporoparietal junction. J. Neurosci. 25, 550–557. 10.1523/JNEUROSCI.2612-04.2005 15659590 PMC6725328

[B18] Blanke O. Ortigue S. Landis T. Seeck M. (2002). Neuropsychology: stimulating illusory own-body perceptions. Nature 419, 269–270. 10.1038/419269a 12239558

[B19] Bos E. M. Spoor J. K. H. Smits M. Schouten J. W. Vincent A. J. P. E. (2016). Out-of-body experience during awake craniotomy. World Neurosurg. 92, 586.e9–586.e13. 10.1016/j.wneu.2016.05.002 27178238

[B20] Bova M. (2011). Recollections of Jimmy's out-of-body experiences. NeuroQuantology 9, 526–529. 10.14704/nq.2011.9.3.461

[B21] Brandt C. Kramme C. Storm H. Pohlmann-Eden B. (2009). Out-of-body experience and auditory and visual hallucinations in a patient with cardiogenic syncope: Crucial role of cardiac event recorder in establishing the diagnosis. Epilepsy Behav. 15, 254–255. 10.1016/j.yebeh.2009.02.047 19268716

[B22] Brumblay R. (2003). Hyperdimensional perspectives in out-of-body and near-death experiences. J. Near-Death Stud. 21, 201–221. 10.1023/A:1024054029979

[B23] Campillo-Ferrer T. Alcaraz-Sánchez A. Demsar E. Wu H.-P. Dresler M. Windt J. Blanke O. (2024). Out-of-body experiences in relation to lucid dreaming and sleep paralysis: a theoretical review and conceptual model. Neurosci. Biobehav. Rev. 163:105770. 10.1016/j.neubiorev.2024.105770 38880408

[B24] Carruthers G. (2018). Confabulation or experience? Implications of out-of-body experiences for theories of consciousness. Theory Psychol. 28, 122–140. 10.1177/0959354317745590

[B25] Chirico A. Pizzolante M. Kitson A. Gianotti E. Riecke B. E. Gaggioli A. (2022). Defining transformative experiences: a conceptual analysis. Front. Psychol. 13:790300. 10.3389/fpsyg.2022.790300 35814064 PMC9263695

[B26] De Foe A. (2012). How should therapists respond to client accounts of out-of-body experience? Int. J. Trans. Stud. 31, 75–82. 10.24972/ijts.2012.31.1.75

[B27] De Foe A. Van Doorn G. Symmons M. (2013). Floating sensations prior to sleep and out-of-body experiences. J. Parapsychol. 77, 271–280.

[B28] De Ridder D. Van Laere K. Dupont P. Menovsky T. Van de Heyning P. (2007). Visualizing out-of-body experience in the brain. N. Engl. J. Med. 357, 1829–1833. 10.1056/NEJMoa070010 17978291

[B29] Facco E. Casiglia E. Al Khafaji B. E. Finatti F. Duma G. M. L. Mento G.. (2019). Neurophenomenology of out-of-body experiences induced by hypnotic suggestions. Int. J. Clin. Exp. Hypn. 67, 1–30. 10.1080/00207144.2019.1553762 30702402

[B30] Fang T. Yan R. Fang F. (2014). Spontaneous out-of-body experience in a child with refractory right temporoparietal epilepsy: case report. J. Neurosurg. 14, 396–399. 10.3171/2014.6.PEDS13485 25062304

[B31] Gabbard G.O. Twemlow S.W. (1984). With the Eyes of the Mind: An Empirical Analysis of Out-Of-Body States. New York: Praeger.

[B32] Gallo F. T. Spiousas I. Herrero N. L. Godoy D. Tommasel A. Gasca-Rolin M. Ramele R. Gleiser P. M. Forcato C. (2023). Structural differences between non-lucid dreams, lucid dreams and out-of-body experience reports assessed by graph analysis. Sci. Rep. 13:19579. 10.1038/s41598-023-46817-2 37949978 PMC10638299

[B33] Gow K. Lang T. Chant D. (2004). Fantasy proneness, paranormal beliefs and personality features in out-of-body experiences. Contemp. Hypn. 21, 107–125. 10.1002/ch.296

[B34] Hameroff S. Penrose R. (2014). Consciousness in the universe: a review of the 'Orch OR' theory. Phys. Life Rev. 11, 39–78. 10.1016/j.plrev.2013.08.002 24070914

[B35] Husserl E. (1931). Ideas: General Introduction to Pure Phenomenology. Translated by W. R. Boyce Gibson. London: George Allen and Unwin.

[B36] Irwin H. (2000). The disembodied self: an empirical study of dissociation and the out-of-body experience. J. Parapsychol. 64, 261–277.

[B37] Irwin H. J. (1985). Flight of Mind: A Psychological Study of the Out-Of-Body Experience. Metuchen, NJ: Scarecrow Press.

[B38] Irwin H. J. (1988). Out-of-body experiences and attitudes to life and death. J. Am. Soc. Psychical Res. 82, 237–251.

[B39] Klemenc-Ketis Z. (2013). Life changes in patients after out-of-hospital cardiac arrest: the effect of near-death experiences. Int. J. Behav. Med. 20, 7–12. 10.1007/s12529-011-9209-y 22161220

[B40] Lopez C. Elzière M. (2018). Out-of-body experience in vestibular disorders: a prospective study of 210 patients with dizziness. Cortex 104, 193–206. 10.1016/j.cortex.2017.05.026 28669509

[B41] Metzinger T. (2005). Out-of-body experiences as the origin of the concept of a “soul”. Mind Matter, 3, 57–84.

[B42] Mudgal V. Dhakad R. Mathur R. Sardesai U. Pal V. (2021). Astral projection: a strange out-of-body experience in dissociative disorder. Cureus 13:e17037. 10.7759/cureus.17037 34522516 PMC8425774

[B43] Murray C. D. Fox J. (2005). Dissociational body experiences: differences between respondents with and without prior out-of-body-experiences. Br. J. Psychol. 96, 441–456. 10.1348/000712605X49169 16248935

[B44] Neppe V. M. (2011). Models of the out-of-body experience: a new multi-etiological phenomenological approach. NeuroQuantology 9, 72–83. 10.14704/nq.2011.9.1.391

[B45] O'Brien B. J. Harris I. B. Beckman T. J. Reed D. A. Cook D. A. (2014). Standards for reporting qualitative research: a synthesis of recommendations. Acad. Med. 89, 1245–1251. 10.1097/ACM.0000000000000388 24979285

[B46] Osis K. (1979). “Insiders' views of the OBE: a questionnaire survey,” in Research in Parapsychology, ed. W. G. Roll (Metuchen, NJ: Scarecrow Press), 50–52.

[B47] Pace J. C. Drumm D. L. (1992). The phantom leaf effect and its implications for near-death and out-of-body experiences. J. Near-Death Stud. 10, 233–240. 10.1007/BF01074166

[B48] Palmer J. (1979). A community mail survey of psychic experiences. J. Am. Soc. Psychical Res. 73, 221–251.

[B49] Parra A. (2008). Out-of-body experiences and hallucinatory experiences: relationship with cognitive and perceptual variables. Liberabit 14, 5–14.

[B50] Parra A. (2010). Out-of-body experiences and hallucinatory experiences: a psychological approach. Imagination Cogn. Pers. 29, 211–223. 10.2190/IC.29.3.d34522516

[B51] Parra A. (2018). Out of body experiences: an evaluation of the construct of transliminality and “thin” boundaries as cognitive-perceptual anomaly. Psicol. Conocimiento Soc. 8, 100–116.

[B52] Persinger M. A. (2010). The harribance effect as pervasive out-of-body experiences: NEUROQUANTAL evidence with more precise measurements. NeuroQuantology, 8, 444–465. 10.14704/nq.2010.8.4.301

[B53] Rabeyron T. Caussié S. (2016). Clinique des sorties hors du corps: trauma, réflexivité et symbolisation, L'Evolution Psychiatrique 81, 755–775. 10.1016/j.evopsy.2015.10.007

[B54] Raduga M. Kuyava O. Sevcenko N. (2020). Is there a relationship between REM sleep dissociated phenomena, like lucid dreaming, sleep paralysis, out-of-body experiences, and false awakening? Med. Hypotheses, 144:110169. 10.1016/j.mehy.2020.110169 32795836

[B55] Ring K. Cooper S. (1999). Mindsight: Near-Death and Out-of-Body Experiences in the Blind. Palo Alto, CA: William James Center for Consciousness Studies.

[B56] Roisin J. (2009). La sortie du corps et autres expériences extrêmes en situation de traumatisme. Rev. Francophone Stress Trauma 9, 71–79.

[B57] Sellers J. (2017). Out-of-body experience: Review and a case study. J. Conscious. Explor. Res. 8, 686–708.

[B58] Sellers J. (2018). A brief review of studies of out-of-body experiences in both the healthy and pathological populations. J. Cogn. Sci. 19, 471–491. 10.17791/jcs.2018.19.4.471

[B59] Shaw J. Gandy S. Stumbrys T. (2023). Transformative effects of spontaneous out of body experiences in healthy individuals: An interpretative phenomenological analysis. Psychol. Conscious.: Theory Res. Pract. Advance online publication. 10.1037/cns0000324

[B60] Smith A. M. Messier C. (2014). Voluntary out-of-body experience: an fMRI study. Front. Hum. Neurosci. 8, 1–10. 10.3389/fnhum.2014.00070 24575000 PMC3918960

[B61] Smith J. A. Flowers P. Larkin M. (2009). Interpretative Phenomenological Analysis: Theory, Method and Research. London: SAGE.

[B62] Terhune D. B. (2006). Dissociative alterations in body image among individuals reporting out-of-body experiences: a conceptual replication. Percept. Motor Skills 103, 76–80. 10.2466/pms.103.1.76-80 17037645

[B63] Thakkar K. N. Nichols H. S. McIntosh L. G. Park S. (2011). Disturbances in body ownership in schizophrenia: evidence from the rubber hand illusion and case study of a spontaneous out-of-body experience. PLoS ONE 6:e27089. 10.1371/journal.pone.0027089 22073126 PMC3205058

[B64] Tononi G. Koch C. (2015). Consciousness: here, there and everywhere? Philos. Trans. R. Soc. B. 370, 1–18. 10.1098/rstb.2014.0167 25823865 PMC4387509

[B65] Tressoldi P. E. Pederzoli L. Caini P. Ferrini A. Melloni S. Prati E.. (2015). Hypnotically induced out-of-body experience: How many bodies are there? Unexpected discoveries about the subtle body and psychic body. Sage Open 5. 10.1177/2158244015615919

[B66] Twemlow S. W. (1989). Clinical approaches to the out-of-body experience. J. Near-Death Stud. 8, 29–43. 10.1007/BF01076137

[B67] Van Lommel P. Wees R. Meyers V. Elfferich I. (2001). Near-death experience in survivors of cardiac arrest: aprospective study in the Netherlands. Lancet 358, 2039–2045. 10.1016/S0140-6736(01)07100-8 11755611

[B68] Weiler M. Acunzo D. J. (2024). What out-of-body experiences may tell us about the mind beyond the brain. Int. Rev. Psychiatry 110. 10.1080/09540261.2024.2436598 40627507

[B69] Wilde D. Murray C. D. (2009). An interpretive phenomenological analysis of out-of-body experiences in two cases of novice meditators. Aust. J. Clin. Exp. Hypn. 37, 90–118.

[B70] Wilde D. Murray C. D. (2010). Interpreting the anomalous: finding meaning in out-of-body and near-death experiences. Qual. Res. Psychol. 7, 57–72. 10.1080/14780880903304550

[B71] Wilkins L. K. Girard T. A. Cheyne J. A. (2011). Ketamine as a primary predictor of out-of-body experiences associated with multiple substance use. Conscious. Cogn. 20, 943–950. 10.1016/j.concog.2011.01.005 21324714

[B72] Wu H. P. Nakul E. Betka S. Lance F. Herbelin B. Blanke O. (2023). Out-of-body illusion induced by visual-vestibular stimulation. iScience 27:108547. 10.1016/j.isci.2023.108547 38161418 PMC10755362

[B73] Yu K. Liu C. Yu T. Wang X. Xu C. Ni D.. (2018). Out-of-body experience in the anterior insular cortex during the intracranial electrodes' stimulation in an epileptic child. J. Clin. Neurosci. 54, 122–125. 10.1016/j.jocn.2018.04.050 29793775

[B74] Zingrone N. L. Alvarado C. S. Cardeña E. (2010). Out-of-body experiences and physical body activity and posture: responses from a survey conducted in Scotland. J. Nerv. Ment. Dis. 198, 163–165. 10.1097/NMD.0b013e3181cc0d6d 20145494

